# Modeling the Antileukemia Activity of Ellipticine-Related Compounds: QSAR and Molecular Docking Study

**DOI:** 10.3390/molecules25010024

**Published:** 2019-12-19

**Authors:** Edgar Márquez, José R. Mora, Virginia Flores-Morales, Daniel Insuasty, Luis Calle

**Affiliations:** 1 Grupo de Investigación en Química y Biología, Departamento de Química y Biología, Universidad del Norte, Cra 51B, Km 5, vía Puerto Colombia, Barranquilla 081007, Colombia; insuastyd@uninorte.edu.co; 2 Grupo de Química Computacional y Teórica (QCT-USFQ) & Instituto de Simulación Computacional (ISC-USF), Departamento de Ingeniería Química, Colegio Politécnico de Ciencias e Ingeniería, Diego de Robles, y vía Interoceánica, Universidad San Francisco de Quito, Quito 170901, Ecuador; 3 Laboratorio de Síntesis Asimétrica y Bioenergética (LSAyB), Ingeniería Química (UACQ), Program of Doctorate in Sciences with orientation in Molecular Medicine, Academic Unit of Human Medicine and Health Sciences, Universidad Autónoma de Zacatecas, Campus XXI Km 6 Carr. Zac-Gdl Edificio 6, 98160 Zacatecas, Mexico; 4 Instituto de Salud Integral (ISAIN), Facultad de Medicina, Universidad Católica Santiago de Guayaquil, Guayaquil 09013493, Ecuador; luis.calle02@cu.ucsg.edu.ec

**Keywords:** cancer, leukemia, molecular modeling, QSAR, molecular descriptors

## Abstract

The antileukemia cancer activity of organic compounds analogous to ellipticine representes a critical endpoint in the understanding of this dramatic disease. A molecular modeling simulation on a dataset of 23 compounds, all of which comply with Lipinski’s rules and have a structure analogous to ellipticine, was performed using the quantitative structure activity relationship (QSAR) technique, followed by a detailed docking study on three different proteins significantly involved in this disease (PDB IDs: SYK, PI3K and BTK). As a result, a model with only four descriptors (HOMO, softness, AC1RABAMBID, and TS1KFABMID) was found to be robust enough for prediction of the antileukemia activity of the compounds studied in this work, with an R^2^ of 0.899 and Q^2^ of 0.730. A favorable interaction between the compounds and their target proteins was found in all cases; in particular, compounds **9** and **22** showed high activity and binding free energy values of around −10 kcal/mol. Theses compounds were evaluated in detail based on their molecular structure, and some modifications are suggested herein to enhance their biological activity. In particular, compounds **22_1**, **22_2**, **9_1**, and **9_2** are indicated as possible new, potent ellipticine derivatives to be synthesized and biologically tested.

## 1. Introduction

Cancer disease represents one of the most significant health problems in the world, being the second most common cause of death around the planet [1,2]. It is estimated that by 2030, the number of cancer cases in the world will have increased by approximately 23.6 million [2]. The situation in the Latin American population is even more dramatic; according to the World Health Organization (WHO) data, 37% of cancer cases are reported in this region, and are associated with the limited development of technology and industry related to the treatment of this disease in these countries [3]. Childhood cancer is considered a rare disease, with between 0.5% and 3% of the malign neoplasms reported around the world found in children. Nevertheless, this cancer type represents a significant public health problem due to the high probability of death in children, which generates a tremendous social impact on the families [4].

In this respect, leukemia is the principal childhood cancer. For children under 15, this type of cancer has a 30% rate of morbidity. Moreover, it seems that this kind of cancer affects more children living in poorer countries. In Latin America, the statistics is alarming: in Colombia, leukemia is responsible for around 49% of cancer deaths, while in Mexico, this kind of cancer is responsible for 22% of childhood deaths [5]. In contrast, in industrially and technologically developed countries, the picture that emerges is encouraging: survival in leukemia patients has increased notably in the last years. Around 90% survival rates are reported in countries such as the United States and the United Kingdom. According to these statistics, 10% of patients were non-responsive to primary chemotherapy and consequently died [4,5,6].

Nowadays, a major concern in public health is the quality of life of childhood leukemia patients, especially related the side effects and toxicity of the drugs used to treat this kind of disease. In this regard, studies of leukemia biology have increased in the past decade [7,8]. Some recent articles have reported the discovery of protein targets that play an essential function in the life cycle of leukemia cells. This knowledge represents a subtle but real thread of hope in the search for new and novel compounds with a high affinity for this target; additionally, understanding the action mechanisms of thse targets could help researchers to improve the activity and toxicity of cancer drugs [9,10,11,12].

In recent decades, quantitative structure–activity relationships (QSAR) are among the techniques most often used to generate new, promising compounds against diseases such as malaria [13], diabetes [14], and cancer [15], among others. By definition, a QSAR model is an equation that involves molecular descriptors with a remarkable influence on a particular biological activity. The knowledge of these chemical characteristics will allow the development of new compounds with better activity and better therapeutic indexes. Moreover, it has been shown that the QSAR-based synthesis has a high probability of success in any disease [16,17].

At the same time, another technique widely used in the development of new drugs is molecular docking [18,19]. This tool has become more and more critical in drug design because of the extraordinary advances in protein purification, nuclear magnetic resonance in proteins, and protein crystallography, which have contributed to elucidating the structural details in ligand–protein complexes. This technique can be used to model the interactions between small molecules and any protein at the atomic scale, allowing researchers to describe the behaviors of these small molecules at the binding site (active site).

The knowledge of both a leader structure from QSAR and its interaction type at the active site of the protein target from docking techniques represents a promising path in the search for and the development of new series of molecules active against a particular disease [20,21,22,23,24,25,26].

Regarding leukemia, the literature reveals extensive effort paid to the search for new molecules with potential applications in the treatment this disease. QSAR techniques have been applied to several families of compounds to find the structural characteristics that influence antileukemia activity [26,27,28]. More recently, some works have reported relationships between the structures of a compound family and affinity with a particular protein that plays a fundamental role in leukemia cell growth [29,30,31,32].

Ellipticine is a natural metabolite from the plant *Ochrosia elliptica* (Apocynaceae), and it has a potent anticancer activity. Its mechanism of action is related to DNA intercalation or via the inhibition of topoisomerase II protein [33,34,35]. Besides the significant activity of this compound, it is not yet available in the pharmaceutical market because of several side effects like nausea, vomiting, hypertension, and fatigue. We believe that strategies to eliminate or minimize the adverse effects of ellipticin can be derived based on functionalization, structural modification, or, more drastically, the search for new compounds analogous to this nucleus.

Among the compounds similar to ellipticine are the benzodioxinic analogues. Some studies have reported that the presence of oxygen atoms as a cyclic peroxide (dioxygen) or as an ester group on the ellipticine moiety seems to have a significant influence on the compound’s biological activity [36,37,38,39]. The structural similarity with ellipticine is, in theory, a fundamental key to designing new anticancer compounds. Moreover, the vast quantity of reported compounds with structures close to ellipticine represents a unique opportunity to identify the common structural characteristic that any molecular structure must have to be active against leukemia. In this respect, this work aimed to find a quantitative relationship between several molecular descriptors (topological, thermodynamics, and electronics) and the antileukemia activity of compounds related to ellipticine, in order to guide the synthesis of new promising antileukemia compounds. Additionally, to offer more insight into the interaction of ellipticine derivatives with leukemia cells, a docking calculation on the selected molecular target of the L1210 leukemia line cell is presented.

## 2. Results

**Figure 1:**
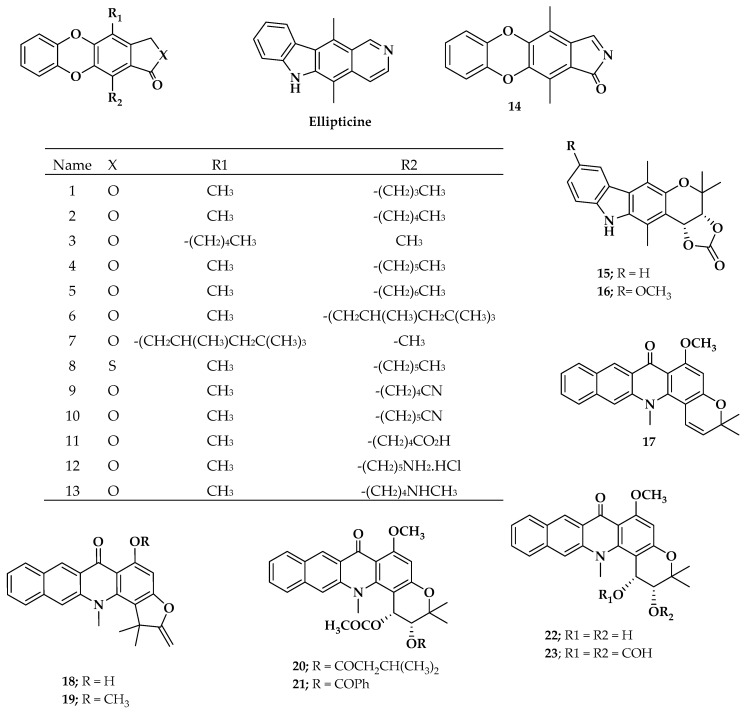
Chemical structures of ellipticine-analogous compounds studied in this work.

Pharmacological data in vitro of several ellipticine analogues with antileukemia activity against L1210 cells were collected from the literature [40,41]. After the application of the Lipinski [42] rule filters, only 23 ellipticine analogues (ellipticine include) were selected. Figure 1 shows the chemical structure of the compounds studied herein.

### 2.1. Molecular Modeling

The minimum-energy 3D geometries for the compounds shown in Figure 1 were obtained using density functional theory with WB97XD/6-311G(d,p) as a theory level [43], using Gaussian 16 software [44] for Linux available in the high performing computer of the San Francisco de Quito University, Quito, Ecuador. The DFT level and interchange correlation functional was chosen because of its good correlation with experimental results based on the energetics and structure of organic molecules [45,46,47,48].

The minimum geometry structure was verified using the second derivative criteria [49]. In this regard, the vibrational frequency calculations were performed for the entire dataset and displayed no imaginary frequency, indicating that all of the geometries were minimum-geometry structures. Both minimum structures and frequency calculations were used to find electronic and molecular descriptors such as dipolar momentum (μ), HOMO (High Occupied Molecular Orbital) and LUMO (Low Unoccupied Molecular Orbital) energies, polarizability (α), enthalpy (H), entropy (S), free energy (G), ionization potential (PI), electronic affinity energy (EAE), hardness (η), softness (s), electrophilic index (ω), lipophilia (ClogP), polar surface area (PSA), topological index (TI), Balaban index (BI), hydrogen bond acceptor (HA), hydrogen bond donator (HD), AC[1]_K_F_AB_nCi_2_M1_NS0_C_LGL[8-9]_a_MID (AC1RABABMID), and TS[1]_K_F_AB_nCi_2_M1_SS0_T_LGL[2-3]_a_MID (TS1KFABMID). Except for AC1RABABMID and TS1KFABMID, these topological indexes were computed using Chemaxon [50], while AC1RABABMID and TS1KFABMID were calculated using QuBiLs-MIDAS as reported previously [51].

### 2.2. Statistical Analysis

The linear regression (LR) method was employed to find the most relevant molecular descriptors—the parameters related to the biological activity. Thus, the biological activity as pIC_50_ (−ln(1/IC_50_), dependent variable) was plotted against each molecular descriptor. A regression coefficient > 0.5 was considered to indicate an “important” molecular descriptor.

According to the linear regression analysis (LRA), Table 1 reveals that four electronic molecular descriptors (HOMO, softness, LUMO, and dipolar momentum (μ)) and three topological molecular descriptors (AC1RABABMID, TS1KFABMID, and PSA) have a strong influence over biological activity.

#### QSAR Model

Using the descriptors shown in Table 1 as independent variables, we obtained the mathematical models described by Equations (1)–(3), with three–four descriptors.

pIC_50_ = 6.3074 + 1.2938 s + 19.794 AC1RABABMID + 1.5161 TS1KFABMID R^2^ = 0.798, F = 25.0, s = 1.023, Q^2^ = 0.650, a(R^2^) = 0.064, a(Q^2^) = −0.453 (1)

pIC_50_ = 12.533 − 0.0189 PSA + 18.828 AC1RABABMID + 1.6302 TS1KFABMID R^2^ = 0.652, F = 11.9, s = 1.342, Q^2^ = 0.506, a(R^2^) = 0.036, a(Q^2^) = −0.287 (2)

pIC_50_ = 20.530 + 51.942 HOMO + 20.763 AC1RABABMID + 1.5447 TS1KFABMID + 1.4174 s R^2^ = 0.836, F = 23.0, s = 0.946, Q^2^ = 0.729, a(R^2^) = 0.126, a(Q^2^) = −0.505 (3)

**Figure 2:**
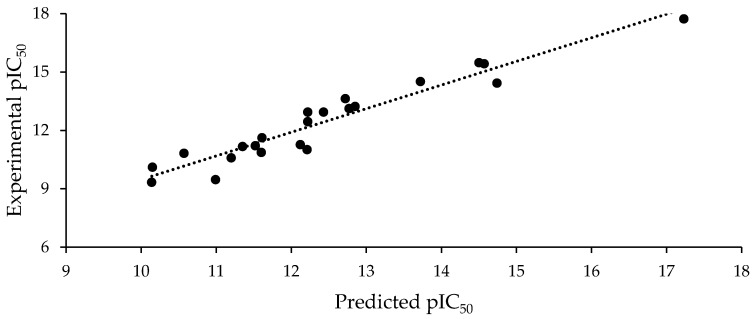
Experimental pIC_50_ vs. pIC_50_ predicted using Equation (3).

The best model, selected according to the statistical robustness, was Model 3, with an R^2^ of 0.836, a high Fisher ratio value of 27.02, and a great correlation prediction index (Q^2^). A Y-scrambling analysis was also performed on Model 3, and found values of a(R^2^) = 0.126 and a(Q^2^) = −0.505, which suggest that the models predictability cannot be explained by chance. The correlation prediction index was estimated by using the leave-one-out crossvalidation. Figure 2 graphically represents the linear relationship between the experimental pIC_50_ values and those predicted using Equation (3).

The correlation matrix of Model 3 is shown in Table 2. Note that the Pearson’s correlation coefficient in each descriptor was <0.6, indicating that the model did not over fit. The descriptors AC1RABABMID and TS1KFABMID seemed to have the most influence over the activity.

According to Equation (3), the highest occupied molecular orbital, HOMO, has a large influence over the antileukemia activity. Thus, the more negative the HOMO energy, the greater the impact on biological activity. This descriptor is related to the molecule’s capability to participate in dipole/dipole interactions like hydrogen bonds. Additionally, the HOMO energy describes the ionization potential and the molecule’s vulnerability to electrophiles attack. HOMO energy also plays a pivotal role in free radical reactions and redox potential [52,53,54,55] therefore, it is very common to find this descriptor in works related to anticancer activity.

The global softness (s) related to the antileukemia activity shown for the compounds studied herein. Global softness is defined as the reciprocal of global hardness and describes the extent to which the electronic environment surrounding the nucleus/nuclei of an atomic/molecular species tends to loosen itself [56,57,58,59,60]. In this sense, it could be associated with a compound’s ability to deform its electronic cloud via variation of the number of electrons. Therefore, a soft compound necessarily has a high potential to transfer electrons in a redox process. As reported by Table 1, ellipticine had the highest softness value, while the rest of the studied compounds had values between 3 and 5, with the most actives ones values close to 5.

The topographic descriptors AC1RABABMID and TS1KFABMID, which were estimated using QuBiL-MIDAS software, have been widely used for the construction of QSAR models for several biological activities [51,61]. These descriptors presented a good correlation with the pIC_50_ values, as shown in Table 2, and they were obtained by the application of some algebraic linear indexes, considering an autocorrelation and a total sum invariant with a lag value of 1 (AC1 and TS1) on the 3D optimized structure. These two descriptors were atomic weighted by the physicochemical property logP, denoted with “a” at the end of the descriptor, and this property is related to the water solubility as well as to the lipophilicity of the compounds, which both play an important role in almost any biological activity of an organic compound.

### 2.3. Molecular Docking

A molecular docking tool was used to gain more insight into the binding modes of the ellipticine-related compounds studied herein with the selected targets. It is necessary to mention that with the exception of ellipticine, the mechanisms of antileukemial cell proliferation inhibition by the compounds studied herein are not well known. One author proved that, in contrast to ellipticine, these kind of compounds are not able to inhibit topoisomerases I and II proteins [40,41]. In this regard, it seems that the antileukemial activity might be associated with other mechanisms that involve different targets than topoisomerases.

According to the literature, spleen tyrosine kinase (SYK), phosphoinositide 3’kinase (PI3K), and Bruton’s tyrosine kinase (BTK) were selected because they have been related to decrease of leukemia cells [62,63,64] in preclinical models. Morover, some compounds structurally close to those studied herein have been reported as SYK [65], BTK [66], and PI3K inhibitors [67]. In this regard, the entire dataset shown in Table 3 was docked against the three targets mentioned above. Due to the inactivity against topoisomerase II, this target was ruled out.

Fostamatinib (R788) is one of the latest reported inhibitors of both SYK and PI3K leukemia proteins; its action mechanism relates to the blocking of antigen-dependent B-cell-receptor signaling [66,67]; thus, based on its already known antileukemial activy, it was used as the control ligand in the docking study.

Table 3 reveals some interesting patterns: the most active compounds (pIC_50_ > 13) showed scoring values smaller than −8 kcal/mol against any target. It is also of note that the less active compounds (pIC_50_ < 11) had at least one value scored higher than −8 kcal/mol. A close inspection of Table 3 reveals that compounds **9**, **20**, and **22** can be highlighted because of their scoring values close to those of compound R788.

**Figure 3:**
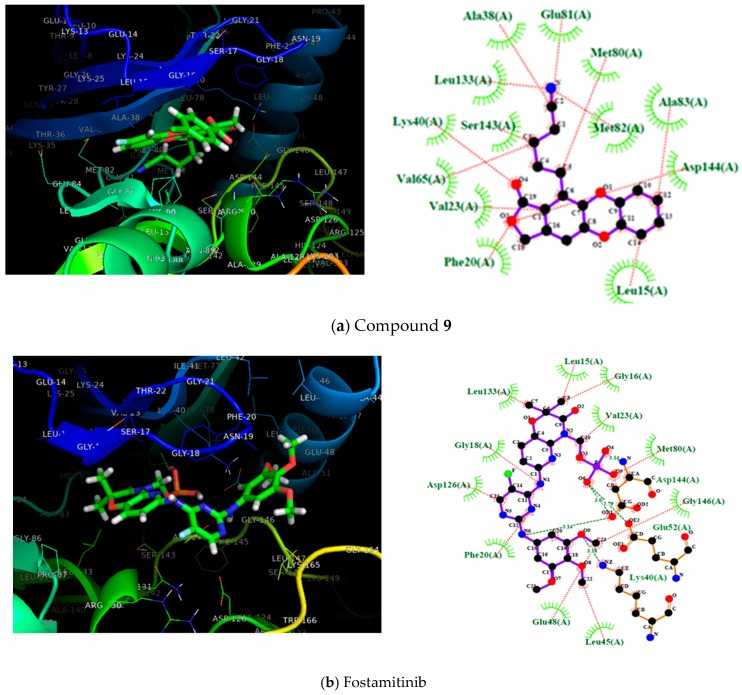

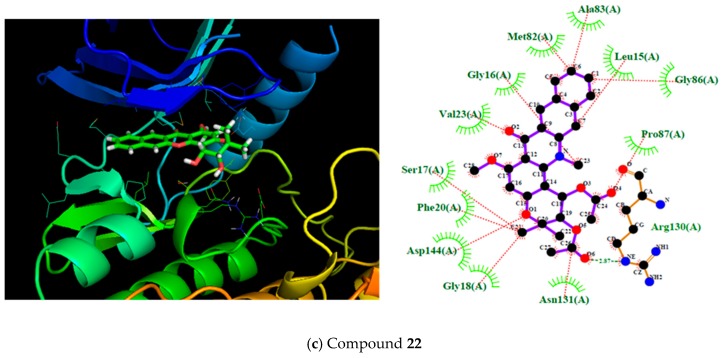
On the left, the 3D representation is shown, and on the right, the 2D interactions of compound 9 (**a**), fostamitinib (**b**), and compound 22 (**c**) at the active site of spleen tyrosine kinase (SYK).

According to Table 1, the entire dataset could be divided into two structural groups; compound **13** derivatives and compound **22** derivatives. Compounds **9** and **22** had the highest antileukemia activity and the highest scoring values into their group. Thus, to gain more details about these two compounds, Figure 3 shows these molecules docked with the proteins SYK, PI3K, and BTK. However, because only the SYK protein has a reported inhibitor, this protein was selected to show the interactions with **9** and **22** compared with that inhibitor (fostamatinib). Thus, the molecular interaction between these ligands and SYK protein is shown in Figure 3; this was generated using Pymol [68] and ligplot software [69].

Figure 3 shows the most active compounds (**9** and **22**) and the structure of fostamitinib with the target protein, as well as a 2D diagram of interactions with the terminal residues of the protein. Further, the figure shows the pose of compounds **9** and **22** bound to the SYK protein; the inhibitor R788 (fostamatinib) is also displayed. It can be noted that compounds **9**, **22**, and fostamatinib contact with the protein via the same pocket. This result is fascinating because it suggests that these compounds might act via a similar mechanism. However, some differences were found in the molecular interaction types.

As shown in Figure 3, the molecular interactions between SYK and fostamatinib were mostly, dipolar in nature. The figure highlights the hydrogen bonds between Glu48 and Leu45 with the OH fragment, Lys40 and Gly18 with NH, and Met80 with the P–OH fragment in fostamatinib. In contrast, compound **9** did not present hydrogen bond formation with the target; however, other dipolar interactions, such as LEU133, Met82, and Glu81 with the CN aliphatic chain, and hydrophobic interactions such as cation–pi (Lys40) or pi–pi (PHE20), did take place.

Compound **22** showed a similar behavior to fostamatinib. This compound displayed several hydrogen bonds formed with the residues ASN131 and ARG130. Additionally, other dipolar interactions were noted, such as with PRO87, Asp144, and Val23. The rest of the interactions were in the hydrophobic range.

**Figure 4:**
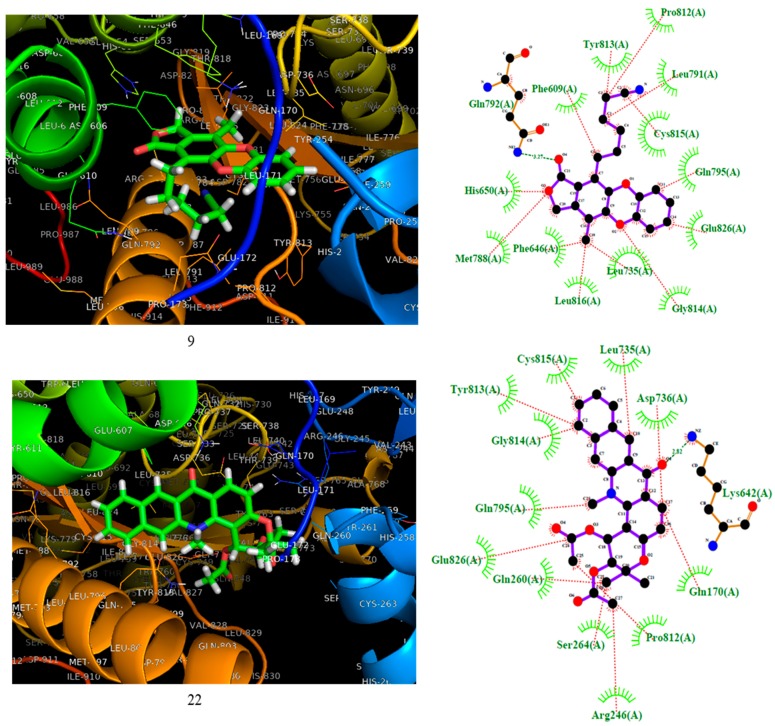
On the left, the 3D representation is shown, and on the right the 2D interactions of compounds **9** and **22** at the active site of PI3K.

Compounds **9** and **22** were docked against phosphoinositide 3’kinase (PI3K). The 3D and 2D docking interaction diagrams are shown in Figure 4. As shown in Figure 4, both compounds **9** and **22** hit the PI3K protein at the same pocket as fostamitinib. However, the nature of the interaction was different. While fostamitinib interacts mostly via dipolar interactions (several hydrogen bonds), in general, compound **9**’s activity was related to several hydrophobic interactions, mostly pi interactions; however, a few dipolar interactions could be observed (Hist650, Glu792, and Met788 with CO group). The same behavior was observed for compound **22**. Figure 4 shows that just a dipolar interaction (Lys642 with OCH_3_ group) took place for this compound.

According to the result above, it seems that the selected compounds might hit the proteins SYK and PI3K in the same pocket as the leader compound fostamitinib. This outcome represents a good starting point for the design and testing of these compounds and their derivatives experimentally.

As shown by the QSAR result, antileukemia compound activity demanded a low HOMO energy and high softness. Moreover, a close inspection of the molecular docking results revealed the relationship of scoring value and QSAR descriptors in Model 3: side chains with electronegative substitution drive to more polar molecules with smaller HOMO energy; thus, a better interaction with the molecular target could be.

**Figure 5:**
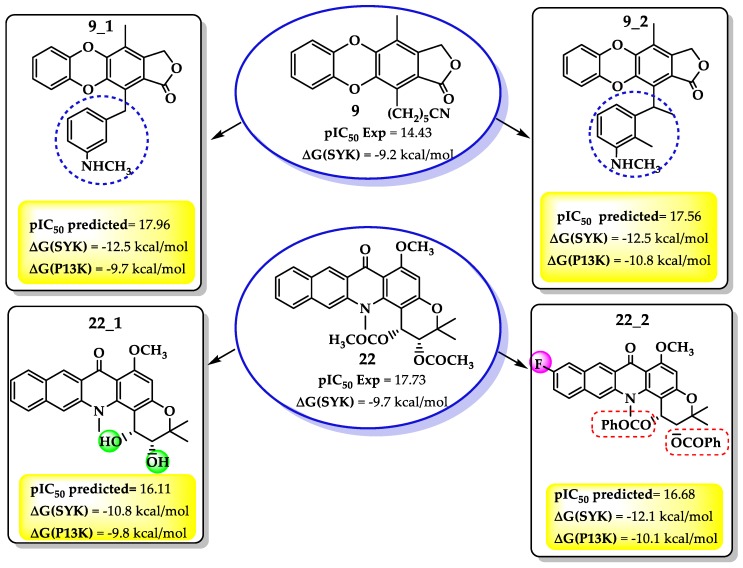
Structures, pIC_50_, and scoring values against SYK protein for new proposed compounds based on QSAR results.

In agreement with the above, i.e., the combination of both molecular behavior and QSAR results, we propose four molecules with the necessary chemical characteristic previously outlined. Figure 5 shows the predicted pIC_50_ and scoring values for these compounds against SYK protein.

As shown in Figure 5, the QSAR model predicted both activity and scoring values higher than its precursor, encouraging the synthesis and experimental evaluation of the proposed compounds. However, as the scoring values for SYK were higher than for PI3K protein, only the docking against SYK was analyzed.

It was noetd that in the compound **9** derivatives, the enhancing of the side-chain with a hydrophobic substituent like a benzene ring increased the antileukemia activity. The benzene ring with an NH terminal not only improved the softness and diminished the HOMO energy, but also enhanced both hydrophobic and dipolar interactions with the selected target. This behavior is highlighted in the SYK proteins when interactions like hydrogen bonds (Arg130 and Asp126) take place.

**Figure 6:**
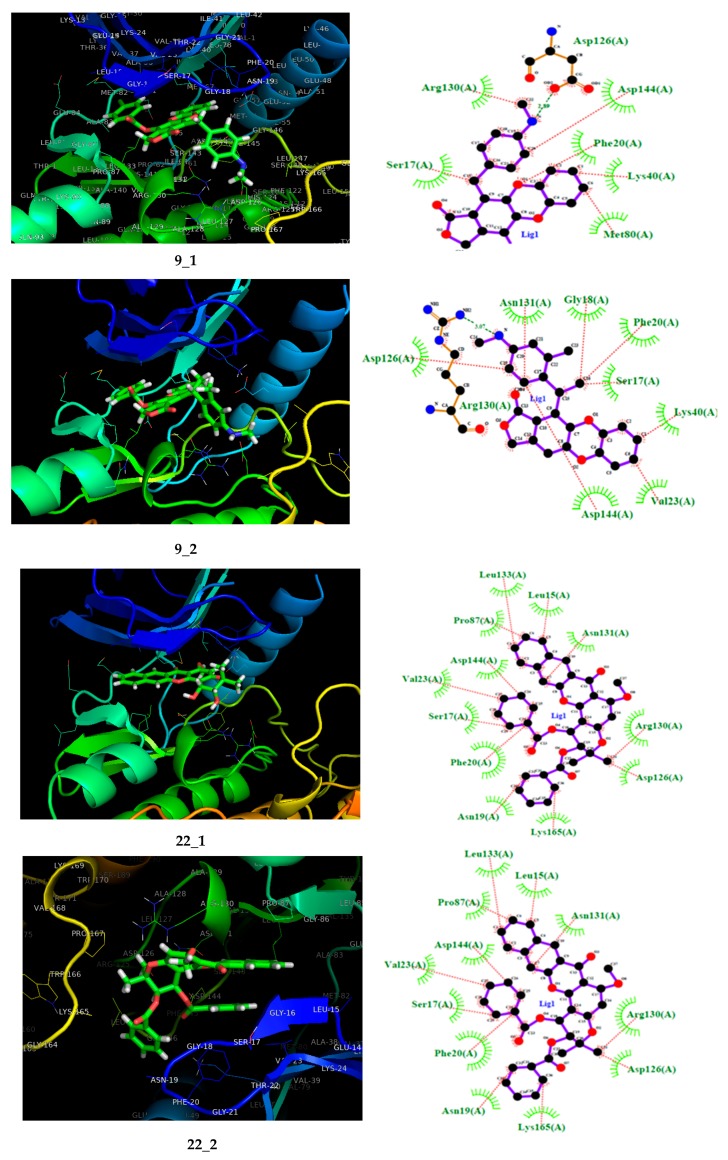
On the left, the 3D representation is shown, and on the right, the 2D interactions of the proposed compound at the spleen tyrosine kinase (SYK) active site.

On the other hand, of the compound **22** derivatives, **22_2** presented the best results against the SYK protein. It was noted that an aromatic ring bound to the ester moiety enhanced the biological activity. Once again, an aromatic ring meets the electronic requirements demands of the QSAR models, i.e., smaller HOMO energy and a higher softness. Additionally, the docking result from SYK target revealed some new interactions driven by further substitutions. For example, as in compound **22**, the hydrogen bonds with ASn131 and Asp130 remain; however, a new hydrogen bond between the ester carbonyl group and Asp144 residue formed. Nevertheless, it seems that the hydrophobic interactions were more critical than for the precursor. Thus, several hydrophobic interactions appeared between the ester moiety aromatic ring and PHE20, Asn19, Val23, Asn19, Lys165, and Ser17 (Figure 6).

The combination of QSAR and docking results in this work seem to support Romero et al.’s idea [41] that the enlargement of the side-chain in ellipticine-related compounds enhance their antileukemia activity. When this enlargement of the side-chain is accompanied by an aromatic ring and electronegative atoms like N, the antileukemia activity is enhanced. Thus, the results reported herein support compounds **9** and **22**. Likewise, their derivatives have a reasonable probability of being active against leukemia cells; therefore, their synthesis and posterior biological testing is encouraged.

**Figure 7:**
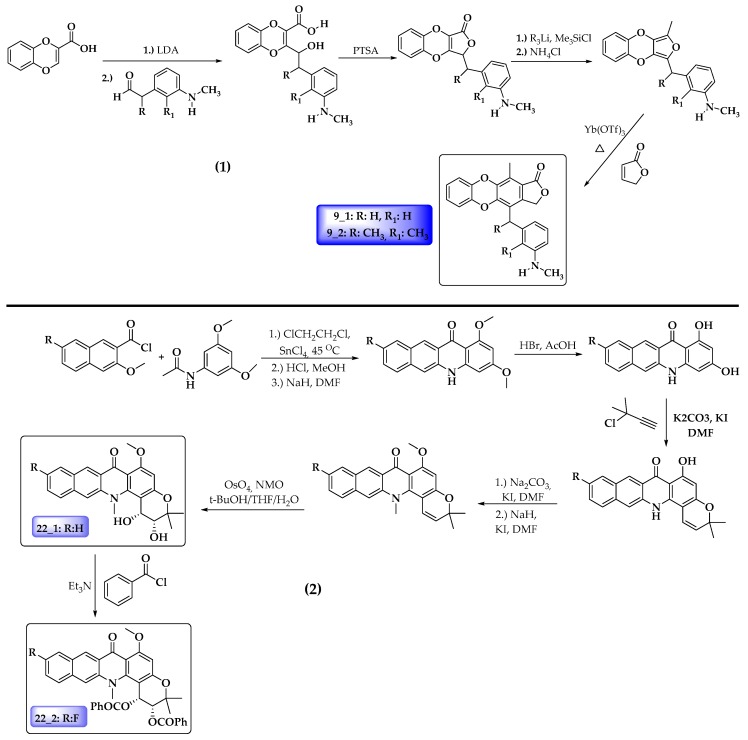
Possible synthetic schema for proposed anticancer compounds. (1) Compounds **9_1** and **9_2**. (2) Compounds **22_1** and **22_2**.

The literature revealed that the synthesis procedure for compound **9_1** has been reported before [66]. However, it was not tested again cancer cells. The rest of the compounds have not been reported to date; however, some similar structure nuclei have been reported. In this regard, based on earlier reports, Figure 7 shows a proposing synthetic route for the four potential antileukemia compounds predicted in this work.

## 3. Materials and Methods

### 3.1. Pharmacological Data Collected

The data of in vitro ability to inhibit 50% L1210 leukemia cell proliferation for 23 ellipticine-related compounds were collected from the literature, mainly from Pujol et al. [40] and Romero et al. [41]. All data collection ensured homogeneity; all of the IC_50_ data were obtained through the same experimental methods. The selected compounds’ IC_50_ values < 100 μM.

### 3.2. Molecular Modeling

The minimum energy geometries for the 23 compounds studied herein were obtained using density functional theory with WB97XD/6-311G(2d,p) as the theory level, using Gaussian 16 software for Linux [44]. Both DFT theory level and interchange correlation functional were chosen because of their good correlation with experimental results based on the energetics and structure of organic molecules [45,46,47].

The minimum geometry structures were verified using the second derivative criteria [50]; the vibrational frequency calculations performed for the entire dataset showed no imaginary frequencies; therefore, all of the geometries were confirmed to be minimum-energy structures. Both the minimum structures and frequency calculations were used to find electronic and molecular descriptors such as dipolar momentum (μ), HOMO and LUMO energies, polarizability (α), enthalpy (H), entropy (S), free energy (G), ionization potential (PI), electronic affinity energy (EAE), hardness (η), softness (s), and electrophilic index (ω) using Equations (4)–(7).

(4) $$\mu =-\chi =\frac{-\left(IP+EA\right)}{2}$$

(5) $$\eta =\frac{\left(IP-EA\right)}{2}$$

(6) $$S=\frac{1}{2\eta }$$

(7) $$\omega =\frac{\mu^{2}}{2\eta }$$

where (µ) is electronic chemical potential and (X) is electronegativity.

The partition coefficient (ClogP), a measure of lipophilicity (a highly influenced descriptor), was obtained using MarvinSketch software for Windows 2017 [50], whereas Gibbs energy, enthalpy, and entropy were obtained by combining frequency calculation and statistical mechanics [49]. The 3D-optimized structures for the all data studied herein were used for the calculation of MAM descriptors, as described previously [14,15]. MAM descriptors are molecular attributes based on n-linear transformation matrix representations weighted over atomic properties like lipophilicity, van der Waals volume (ν), refractivity (r), polar surface area (PSA), polarizability (α), and electronegativity (χ), and these calculations were performed with QuBiLs-MIDAS software [51].

### 3.3. Statistical Analysis

The molecular descriptors were plotted against IC_50_ (as pIC_50_) to find the significance over the biological activity. Descriptors with higher association with antileukemia activity (r > 0.5) were considered statistically relevant and used in the construction of the mathematical model (QSAR).

#### 3.3.1. Quantitative Structure–Activity Relationship (QSAR) and Statistical Validation

The relationships of antileukemia activity and the most relevant molecular descriptors were studied using multiple linear regressions. The method used has been described [13,70] before. Briefly, attempts were made to map the relationship between two or more independent variables with a dependent variable by fitting a linear equation involving the observed data. The independent variables of the model, selected according to forward selection and backward elimination [71,72,73] methods, were three statistical variables: the correlation coefficient (R), the Fisher ratio values (F), and the standard deviation (s).

After the QSAR models were formulated, they were validated statistically before their application in the designs of the new antileukemia molecules. To this end, the predictive power of the best equation was verified via leave-one-out cross-validation methods [74] and quantified by Equation (8). This method has been explained previously in several articles, and is well known for its extensive use in QSAR studies [75,76,77,78].

(8) $$q_{cv}^{2}=1-\frac{\sum_{i=1}^{n}{\left(Y_{exp}-Y_{pred}\right)}^{2}}{\sum_{i=1}^{n}{\left(Y_{exp}-\bar{Y}\right)}^{2}}$$

Likewise, the standard error of prediction (SEP) is calculated as:

(9) $$SEP=\sqrt{\frac{\sum_{i=1}^{n}{\left(yi-\ddot{y}i\right)}^{2}}{n}}$$

where *y* is the experimental value of ln (1/IC_50_), $\ddot{y}$ is the predicted value, and *n* is the number of samples used for model building. A y-scrambling analysis was also performed in order to confirm that the predictability of the models was not due to chance. The a(R^2^) and a(Q^2^) values were then determined using a total of 300 iterations on the response variable, and small values of these correlation coefficients were associated with a correlation not due to chance.

#### 3.3.2. Molecular Docking

The protocol used herein to perform the molecular docking has been reported previously [68,72], and has been used to model the interactions of drugs with the active sites in different diseases, such as Pin1 (peptidyl-prolyl cis-trans isomerase NIMA-interacting 1) [15] inhibition and antimalarial activity [13]. Autodock4 software was used to this end [79]. Tridimensional structures of spleen tyrosine kinase (SYK, PDB-ID:4F4P), phosphoinositide 3´kinase (PI3K, PDB-ID:6DGT), and Buton´s tyrosine kinase (BTK, PDB-ID:3PIY) were obtained from the reported RX in the protein data bank (PDB) as.pdb format [80]. Each protein was refined before use; water molecules and any ligand associated with the protein were removed. Additionally, both polar hydrogen atoms and Kollman-type charge were added. The non-ligand proteins were then saved in .pdb format. Next, the optimized ligands from the WB97XD/6-311G(d,p) theory level were converted to .pdb format and added to the protein. Next, both protein and ligand saved in PDBQT format were used to perform the docking calculation with autodock4 software. The interaction points between ligands and proteins were analyzed using autogrid software, building the grid box into the active center of each protein considering 50 points through the x, y, and z directions, taking into account at least 2.5 million interactions through genetic algorithm, and taking into account a binding site size of 22 Angstrom. The best scoring factors obtained were compared with standard ligands, i.e., the reported inhibitors of the proteins used. These poses were saved in .pdb format and visualized using pymol for Linux. Next, the best pose interaction images were generated using Ligplot; this software allowed us to gain information about the molecular interactions (dipole–dipole and hydrophobic) around the protein active site and the ligand. The identified interactions were validated via the QSAR model.

## 4. Conclusions

The minima energy structures for 23 compounds—including ellipticine, a prominent anticancer compound—were obtained by means of density functional theory, combining the WB97XD method with a 6-311G(d,p) basis set. Multiple linear regression methods were applied over the entire dataset, using both electronic and topological molecular descriptors as independent variables and the pIC_50_ as the dependent variable. After this stepwise approach was used, three mathematical models were generated; according to statistical descriptors, Model 3 had the best results. Moreover, this model was statistically validated through leave-one-out cross-validation methods. According to the results herein, antileukemia activity can be attributed to four molecular descriptors: HOMO, softness, AC1RABAMBID, and TS1KFABMID. These manageable descriptors allowed association of the chemical characteristics with antileukemia activity. The entire dataset was used to perform docking studies with three fundamental protein targets: spleen tyrosine kinase (SYK), phosphoinositide 3’kinase (PI3K), and Bruton’s tyrosine kinase (BTK). Compounds **9** and **22** were highlighted based on their scoring values; moreover, these compounds hit the SYK protein into the same active site as compound R788 (fostamatinib), a reported inhibitor of SYK protein. Furthermore, the scoring values of the four compounds were close to the R788 values. Based on QSAR analysis and the analysis of docking molecular interactions with SYK protein, four compounds are proposed as possible SYK inhibitors; the results achieved herein encourage the synthesis of these four compounds and subsequent biological tests.

## Figures and Tables

**Table 1:** Values of the seven most important properties calculated and their respective values of antileukemial activity. HOMO, softness, and LUMO are expressed in hartree/particle, while μ is expressed in Debye.

Compound	pIC_50_	HOMO	AC1RABABMID	TS1KFABMID	Softness (s)	LUMO	μ	PSA (A)
**1**	12.94	−0.288	0.015	0.762	3.615	0.011	5.636	87.38
**2**	13.23	−0.288	0.007	0.187	4.613	0.011	5.646	91.98
**3**	13.12	−0.288	0.038	0.142	3.614	0.011	5.610	96.58
**4**	12.45	−0.291	0.011	0.197	3.576	0.011	5.575	101.18
**5**	11.21	−0.294	0.010	0.767	3.535	0.011	5.475	105.55
**6**	10.87	−0.288	0.015	0.342	3.502	0.003	5.099	102.75
**7**	9.47	−0.295	0.010	−0.029	3.427	0.003	9.575	73.94
**8**	14.43	−0.291	0.214	−0.199	4.528	0.007	6.154	73.94
**9**	9.33	−0.289	0.022	−0.303	3.612	0.012	6.567	88.38
**10**	11.27	−0.289	0.061	0.036	3.685	0.011	4.510	73.94
**11**	12.94	−0.281	0.022	0.169	4.011	0.015	6.952	69.18
**ELLIPTICINE**	15.48	−0.281	0.095	−1.020	6.100	0.000	3.545	24.06
**12**	13.63	−0.286	0.169	−1.020	3.495	13.630	−0.286	23.59
**13**	13.63	−0.286	0.169	−1.020	3.495	0.007	3.274	30.49
**14**	10.59	−0.274	0.007	−0.193	3.914	0.016	5.912	59.95
**15**	11.01	−0.262	0.055	−0.093	2.046	0.016	6.150	69.18
**16**	10.82	−0.270	0.014	−0.453	3.835	0.007	5.121	41.93
**17**	11.62	−0.287	0.027	0.382	3.369	−0.010	3.638	52.93
**18**	11.17	−0.285	0.046	−0.018	3.410	−0.009	2.842	41.93
**19**	14.51	−0.274	0.019	1.636	3.571	−0.008	7.458	100.85
**20**	15.42	−0.274	0.022	2.150	3.567	−0.008	7.597	100.85
**21**	10.11	−0.278	0.026	−0.660	3.560	−0.003	6.429	82.39
**22**	17.73	−0.288	0.201	2.337	3.292	−0.015	7.236	100.85

**Table 2:** Pearson’s correlation coefficient for the molecular descriptors used in Equation (3).

	pIC_50_	HOMO	AC1RABABMID	TS1KFABMID	Softness
pIC_50_	1	0.012	0.497	0.512	0.210
HOMO		1	−0.151	0.412	0.285
AC1RABABMID			1	−0.010	0.043
TS1KFABMID				1	−0.400
Softness					1

**Table 3:** Scoring values for the entire data studied against the three selected target proteins. SYK: spleen tyrosine kinase; PI3K: phosphoinositide 3’kinase; BTK: Bruton’s tyrosine kinase; R788: fostamatinib.

Compound	pIC_50_	SYK	PI3K	BTK
**1**	12.94	−8.1	−8.3	−9.0
**2**	13.23	−8.9	−9.5	−8.9
**3**	11.84	−8.3	−8.2	−8.9
**4**	13.12	−8.8	−9.1	−9.1
**5**	12.45	−7.6	−8.1	−9.0
**6**	11.21	−7.6	−8.6	−9.7
**7**	10.87	−7.3	−7.5	−7.9
**8**	13.00	−9.5	−9.2	−9.1
**9**	14.43	−9.8	−9.0	−9.8
**10**	9.32	−7.7	−8.4	−8.9
**11**	11.27	−8.8	−8.9	−8.9
**12**	12.94	−8.4	−8.4	−8.8
**Ellipticine**	15.48	−9.5	−9.9	−9.5
**13**	13.63	−9.1	−9.0	−8.4
**14**	10.58	−7.5	−7.4	−10.1
**15**	11.06	−9.0	−10.0	−10.5
**16**	10.83	−7.7	−6.9	−6.8
**17**	11.61	−7.7	−7.9	−7.4
**18**	11.16	−7.5	−9.8	−10.1
**19**	14.51	−8.9	−9.3	−10.1
**20**	15.42	−8.9	−8.7	−8.9
**21**	10.11	−7.7	−7.3	−6.7
**22**	17.73	−9.7	−8.7	−9.7
**R788**	13.23	−9.1	−9.5	−9.4

## References

[1] Song M., Giovannucci E.L. (2015). Cancer risk: Many factors contribute. Science.

[2] Naghavi M., Abajobir A.A., Abbafati C., Abbas K.M., Abd-Allah F., Abera S.F., Aboyans V., Adetokunboh O., Afshin A., Agrawal A. (2017). Global, regional, and national age-sex specific mortality for 264 causes of death, 1980–2016: A systematic analysis for the Global Burden of Disease Study 2016. Lancet.

[3] Facts and Statistics|Leukemia and Lymphoma Society. https://www.lls.org/facts-and-statistics/facts-and-statistics-overview/facts-and-statistics.

[4] Hafez H.A., Soliaman R.M., Bilal D., Hashem M., Shalaby L.M. (2019). Early Deaths in Pediatric Acute Leukemia: A Major Challenge in Developing Countries. J. Pediatr. Hematol. Oncol..

[5] Chen Q., Jain N., Ayer T., Wierda W.G., Flowers C.R., O’Brien S.M., Keating M.J., Kantarjian H.M., Chhatwal J. (2017). Economic burden of chronic lymphocytic leukemia in the era of oral targeted therapies in the United States. J. Clin. Oncol..

[6] Vedi A., Mitchell R., Shanmuganathan S., Oswald C., Marshall G.M., Trahair T., Sivarajasingam S., Ziegler D.S. (2018). Increased Survival for Children With Acute Myeloid Leukemia Results From Improved Postrelapse Treatment. J. Pediatric Hematol. Oncol..

[7] Lins M.M., Santos M.O., Albuquerque M.F.P.M., de Castro C.C.L., Mello M.J.G., de Camargo B. (2017). Incidence and survival of childhood leukemia in Recife, Brazil: A population-based analysis. Pediatric Blood Cancer.

[8] Corella Aznar E.G., Ayerza Casas A., Carboné Bañeres A., Calvo Escribano M.Á.C., Labarta Aizpún J.I., Samper Villagrasa P. (2019). Quality of life and chronic health conditions in childhood acute leukaemia survivors. Med. Clínica.

[9] Giannopoulos K. (2019). Targeting immune signaling checkpoints in acute myeloid leukemia. J. Clin. Med..

[10] Hoseini S.S., Cheung N.K. (2017). Acute myeloid leukemia targets for bispecific antibodies. Blood Cancer J..

[11] Charmsaz S., Al-Ejeh F., Yeadon T.M., Miller K.J., Smith F.M., Stringer B.W., Moore A.S., Lee F.-T., Cooper L.T., Stylianou C. (2017). EphA3 as a target for antibody immunotherapy in acute lymphoblastic leukemia. Leukemia.

[12] Patrussi L., Capitani N., Baldari C.T. (2019). Abnormalities in chemokine receptor recycling in chronic lymphocytic leukemia. Cell. Mol. Life Sci..

[13] Flores-Sumoza M., Alcázar J.J., Márquez E., Mora J.R., Lezama J., Puello E. (2018). Classical QSAR and docking simulation of 4-pyridone derivatives for their antimalarial activity. Molecules.

[14] Mora J.R., Márquez E.A., Calle L. (2018). Computational molecular modelling of N-cinnamoyl and hydroxycinnamoyl amides as potential α-glucosidase inhibitors. Med. Chem. Res..

[15] Cabrera N., Mora J.R., Marquez E.A. (2019). Computational Molecular Modeling of Pin1 Inhibition Activity of Quinazoline, Benzophenone, and Pyrimidine Derivatives. J. Chem..

[16] Roy K., Kar S., Das R.N., Roy K., Kar S., Das R.N. (2015). Chapter 3—Classical QSAR. Understanding the Basics of QSAR for Applications in Pharmaceutical Sciences and Risk Assessment.

[17] Verma J., Khedkar V., Coutinho E. (2010). 3D-QSAR in Drug Design—A Review. Curr. Top. Med. Chem..

[18] Morris G.M., Lim-Wilby M. (2008). Molecular docking. Methods Mol. Biol..

[19] Gupta M., Sharma R., Kumar A. (2018). Docking techniques in pharmacology: How much promising?. Comput. Biol. Chem..

[20] Lushington G.H., Guo J.X., Wang J.L. (2007). Whither Combine? New Opportunities for Receptor-Based QSAR. Curr. Med. Chem..

[21] Talevi A., Gavernet L., Bruno-Blanch L. (2009). Combined Virtual Screening Strategies. Curr. Comput. Aided-Drug Des..

[22] Rasulev B., Leszczynski J. (2016). Recent Developments in 3D QSAR and Molecular Docking Studies of Organic and Nanostructures. Handbook of Computational Chemistry.

[23] Danielson M.L., Hu B., Shen J., Desai P.V., Bhattachar S.N., Morrison J.S., Mudra D.R., Bender D.M. (2017). In Silico ADME Techniques Used in Early-Phase Drug Discovery. Translating Molecules into Medicines.

[24] Halder A.K., Moura A.S., Cordeiro M.N.D.S. (2018). QSAR modelling: A therapeutic patent review 2010–present. Expert Opin. Ther. Pat..

[25] Chen Y.C. (2015). Beware of docking!. Trends Pharmacol. Sci..

[26] Śledź P., Caflisch A. (2018). Protein structure-based drug design: from docking to molecular dynamics. Curr. Opin. Struct. Biol..

[27] Letis A.S., Seo E.J., Nikolaropoulos S.S., Efferth T., Giannis A., Fousteris M.A. (2017). Synthesis and cytotoxic activity of new artemisinin hybrid molecules against human leukemia cells. Bioorganic Med. Chem..

[28] Arthur D.E., Uzairu A., Mamza P., Abechi S.E., Shallangwa G. (2018). Activity and toxicity modelling of some NCI selected compounds against leukemia P388ADR cell line using genetic algorithm-multiple linear regressions. J. King Saud Univ.-Sci..

[29] Zhang L., Chen Y., Liu N., Li L., Xiao S., Li X., Chen K., Luo C., Chen S., Chen H. (2018). Design, synthesis and anti leukemia cells proliferation activities of pyrimidylaminoquinoline derivatives as DOT1L inhibitors. Bioorg. Chem..

[30] Melge A.R., Kumar L.G., Pavithran K., Nair S.V., Manzoor K., Gopi Mohan C. (2019). Predictive models for designing potent tyrosine kinase inhibitors in chronic myeloid leukemia for understanding its molecular mechanism of resistance by molecular docking and dynamics simulations. J. Biomol. Struct. Dyn..

[31] Cheng G., Wang Z., Yang J., Bao Y., Xu Q., Zhao L., Liu D. (2019). Design, synthesis and biological evaluation of novel indole derivatives as potential HDAC/BRD4 dual inhibitors and anti-leukemia agents. Bioorg. Chem..

[32] Furlan V., Konc J., Bren U. (2018). Inverse molecular docking as a novel approach to study anticarcinogenic and anti-neuroinflammatory effects of curcumin. Molecules.

[33] Canals A., Purciolas M., Aymamí J., Coll M. (2005). The anticancer agent ellipticine unwinds DNA by intercalative binding in an orientation parallel to base pairs. Acta Crystallogr. Sect. D Biol. Crystallogr..

[34] Stiborová M., Poljaková J., Martínková E., Bořek-Dohalská L., Eckschlager T., Kizek R., Frei E. (2011). Ellipticine cytotoxicity to cancer cell lines-a comparative study. Interdiscip. Toxicol..

[35] Miller C.M., O’sullivan E.C., McCarthy F.O. (2019). Novel 11-substituted ellipticines as potent anticancer agents with divergent activity against cancer cells. Pharmaceuticals.

[36] Bramhananda Reddy N., Burra V.R., Ravindranath L.K., Naresh Kumar V., Sreenivasulu R., Sadanandam P. (2016). Synthesis and biological evaluation of benzimidazole fused ellipticine derivatives as anticancer agents. Mon. Fur Chem..

[37] Vann K.R., Ergün Y., Zencir S., Oncuoglu S., Osheroff N., Topcu Z. (2016). Inhibition of human DNA topoisomerase IIα by two novel ellipticine derivatives. Bioorganic. Med. Chem. Lett..

[38] Russell E.G., Guo J., O’Sullivan E.C., O’Driscoll C.M., McCarthy F.O., Cotter T.G. (2016). 7-formyl-10-methylisoellipticine, a novel ellipticine derivative, induces mitochondrial reactive oxygen species (ROS) and shows anti-leukaemic activity in mice. Invest. New Drugs.

[39] Madhavi S., Sreenivasulu R., Raju R.R. (2017). Synthesis and biological evaluation of oxadiazole incorporated ellipticine derivatives as anticancer agents. Mon. Fur Chem..

[40] Pujol M., Romero M., Sanchez I. (2005). Synthesis and Biological Activity of New Class of Dioxygenated Anticancer Agents. Curr. Med. Chem. Agents.

[41] Romero M., Renard P., Caignard D.-H., Atassi G., Solans X., Constans P., Bailly C., Pujol M.D. (2007). Synthesis and Structure–Activity Relationships of New Benzodioxinic Lactones as Potential Anticancer Drugs. J. Med. Chem..

[42] Walters W.P. (2012). Going further than Lipinski’s rule in drug design. Expert Opin. Drug Discov..

[43] Chai J.D., Head-Gordon M. (2008). Long-range corrected hybrid density functionals with damped atom-atom dispersion corrections. Phys. Chem. Chem. Phys..

[44] Frisch M.J., Trucks G.W., Schlegel H.B., Scuseria G.E., Robb M.A., Cheeseman J.R., Scalmani G., Barone V., Petersson G.A., Nakatsuji H. (2016). Gaussian16 Revision A.03.

[45] Luiggi M., Mora J.R., Loroño M., Marquez E., Lezama J., Cordova T., Chuchani G. (2014). Theoretical calculations on the gas-phase thermal decomposition kinetics of selected thiomethyl chloroalkanes: A new insight of the mechanism. Comput. Theor. Chem..

[46] Mora J.R., Cervantes C., Marquez E. (2018). New insight into the chloroacetanilide herbicide degradation mechanism through a nucleophilic attack of hydrogen sulfide. Int. J. Mol. Sci..

[47] Lezama J., Márquez E., Mora J.R., Córdova T., Chuchani G. (2009). Theoretical calculations on the mechanisms of the gas phase elimination kinetics of chlorocyclohexane, 3-chlorocyclohexene and 4-chlorocyclohexene. J. Mol. Struct. THEOCHEM.

[48] Marquez E., Domínguez R.M., Mora J.R., Córdova T., Chuchani G. (2010). Experimental and theoretical studies of the homogeneous, unimolecular gas-phase elimination kinetics of trimethyl orthovalerate and trimethyl orthochloroacetate. J. Phys. Chem. A.

[49] McQuarrie D.A. (2000). Statistical Mechanics.

[50] ChemAxon-Software Solutions and Services for Chemistry & Biology. https://chemaxon.com/.

[51] García-Jacas C.R., Marrero-Ponce Y., Acevedo-Martínez L., Barigye S.J., Valdés-Martiní J.R., Contreras-Torres E. (2014). QuBiLS-MIDAS: A parallel free-software for molecular descriptors computation based on multilinear algebraic maps. J. Comput. Chem..

[52] Jungwirth U., Kowol C.R., Keppler B.K., Hartinger C.G., Berger W., Heffeter P. (2011). Anticancer activity of metal complexes: Involvement of redox processes. Antioxid. Redox Signal..

[53] Zhang P., Sadler P.J. (2017). Redox-Active Metal Complexes for Anticancer Therapy. Eur. J. Inorg. Chem..

[54] Blunt C.E., Torcuk C., Liu Y., Lewis W., Siegel D., Ross D., Moody C.J. (2015). Synthesis and Intracellular Redox Cycling of Natural Quinones and Their Analogues and Identification of Indoleamine-2,3-dioxygenase (IDO) as Potential Target for Anticancer Activity. Angew. Chem. Int. Ed..

[55] Novak Jovanović I., Jadreško D., Miličević A., Hranjec M., Perin N. (2019). An electrochemical study on the redox chemistry of cyclic benzimidazole derivatives with potent anticancer activity. Electrochim. Acta.

[56] Geerlings P., De Proft F., Langenaeker W. (2003). Conceptual Density Functional Theory. Chem. Rev..

[57] Sandoval-Yañez C., Mascayano C., Martínez-Araya J.I. (2018). A theoretical assessment of antioxidant capacity of flavonoids by means of local hyper–softness. Arab. J. Chem..

[58] Perea-Ramírez L.I., Guevara-García A., Galván M. (2018). Using local softness to reveal oxygen participation in redox processes in cathode materials. J. Mol. Model..

[59] Willems R.E.M., Weijtens C.H.L., de Vries X., Coehoorn R., Janssen R.A.J. (2019). Relating Frontier Orbital Energies from Voltammetry and Photoelectron Spectroscopy to the Open-Circuit Voltage of Organic Solar Cells. Adv. Energy Mater..

[60] Tagade P.M., Adiga S.P., Park M.S., Pandian S., Hariharan K.S., Kolake S.M. (2018). Empirical Relationship between Chemical Structure and Redox Properties: Mathematical Expressions Connecting Structural Features to Energies of Frontier Orbitals and Redox Potentials for Organic Molecules. J. Phys. Chem. C.

[61] Meneses-Marcel A., Marrero-Ponce Y., Machado-Tugores Y., Montero-Torres A., Pereira D.M., Escario J.A., Nogal-Ruiz J.J., Ochoa C., Arán V.J., Martínez-Fernández A.R. (2005). A linear discrimination analysis based virtual screening of trichomonacidal lead-like compounds: Outcomes of in silico studies supported by experimental results. Bioorganic Med. Chem. Lett..

[62] Davids M.S., Brown J.R. (2012). Targeting the B cell receptor pathway in chronic lymphocytic leukemia. Leuk. Lymphoma.

[63] Woyach J.A., Bojnik E., Ruppert A.S., Stefanovski M.R., Goettl V.M., Smucker K.A., Smith L.L., Dubovsky J.A., Towns W.H., MacMurray J. (2014). Bruton’s tyrosine kinase (BTK) function is important to the development and expansion of chronic lymphocytic leukemia (CLL). Blood.

[64] Burger J.A. (2014). Bruton’s tyrosine kinase (BTK) inhibitors in clinical trials. Curr. Hematol. Malig. Rep..

[65] Weisberg E.L., Puissant A., Stone R., Sattler M., Buhrlage S.J., Yang J., Manley P.W., Meng C., Buonopane M., Daley J.F. (2017). Characterization of midostaurin as a dual inhibitor of FLT3 and SYK and potentiation of FLT3 inhibition against FLT3-ITD-driven leukemia harboring activated SYK kinase. Oncotarget.

[66] Thi Mai H.D., Gaslonde T., Michel S., Tillequin F., Koch M., Bongui J.B., Elomri A., Seguin E., Pfeiffer B., Renard P. (2003). Structure-activity relationships and mechanism of action of antitumor benzo[b]pyrano[3,2-h]acridin-7-one acronycine analogues. J. Med. Chem..

[67] Liu L., Shi B., Li X., Wang X., Lu X., Cai X., Huang A., Luo G., You Q., Xiang H. (2018). Design and synthesis of benzofuro[3,2-b]pyridin-2(1H)-one derivatives as anti-leukemia agents by inhibiting Btk and PI3Kδ. Bioorganic Med. Chem..

[68] Seeliger D., De Groot B.L. (2010). Ligand docking and binding site analysis with PyMOL and Autodock/Vina. J. Comput. Aided. Mol. Des..

[69] Wallace A.C., Laskowski R.A., Thornton J.M. (1995). Ligplot: A program to generate schematic diagrams of protein-ligand interactions. Protein Eng. Des. Sel..

[70] Flores M.C., Márquez E.A., Mora J.R. (2018). Molecular modeling studies of bromopyrrole alkaloids as potential antimalarial compounds: A DFT approach. Med. Chem. Res..

[71] Zhang Z. (2016). Variable selection with stepwise and best subset approaches. Ann. Transl. Med..

[72] Forli S., Huey R., Pique M.E., Sanner M.F., Goodsell D.S., Olson A.J. (2016). Computational protein-ligand docking and virtual drug screening with the AutoDock suite. Nat. Protoc..

[73] Heinze G., Wallisch C., Dunkler D. (2018). Variable selection—A review and recommendations for the practicing statistician. Biometrical. J..

[74] Webb G.I., Sammut C., Perlich C., Horváth T., Wrobel S., Korb K.B., Noble W.S., Leslie C., Lagoudakis M.G., Quadrianto N. (2011). Leave-One-Out Cross-Validation. Encyclopedia of Machine Learning.

[75] Cramer R.D., Bunce J.D., Patterson D.E., Frank I.E. (1988). Crossvalidation, Bootstrapping, and Partial Least Squares Compared with Multiple Regression in Conventional QSAR Studies. Quant. Struct. Relatsh..

[76] Veerasamy R., Rajak H., Jain A., Sivadasan S., Varghese C.P., Agrawal R.K. (2011). Validation of QSAR Models-Strategies and Importance. Int. J. Drug Des. Discov..

[77] Majumdar S., Basak S.C. (2018). Beware of External Validation!—A Comparative Study of Several Validation Techniques used in QSAR Modelling. Curr. Comput. Aided. Drug Des..

[78] Wong T.T. (2015). Performance evaluation of classification algorithms by k-fold and leave-one-out cross validation. Pattern Recognit..

[79] Morris G.M., Ruth H., Lindstrom W., Sanner M.F., Belew R.K., Goodsell D.S., Olson A.J. (2009). Software news and updates AutoDock4 and AutoDockTools4: Automated docking with selective receptor flexibility. J. Comput. Chem..

[80] RCSB PDB: Homepage. https://www.rcsb.org/.

